# Ethical leadership and sustainable employee wellbeing: the roles of emotional energy, moral signals, and psychological resources

**DOI:** 10.3389/fpsyg.2026.1788987

**Published:** 2026-04-09

**Authors:** Wei Chen, Xiaoli Xu, Dan Chen

**Affiliations:** 1 Hubei Communications Technical College, Wuhan, China; 2 Changjiang Institute of Technology, Wuhan, China

**Keywords:** emotional energy, ethical leadership, moral signals, organizational ethics, psychological resources, sustainable wellbeing

## Abstract

Concerns about employee wellbeing have intensified in increasingly demanding and morally complex workplaces, yet existing research offers fragmented explanations of how ethical leadership relates to sustainable psychological functioning. Prior studies frequently conflate short-term affective activation with broader adaptive capacity and rarely integrate leadership behavior and contextual moral signals within a unified analytical structure. Addressing these limitations, this study advances a differentiated psychological framework that analytically separates emotional energy (affective vitality) from psychological resources (relatively stable adaptive capacity) while modeling them simultaneously alongside ethical leadership and moral signals. Using cross-sectional survey data from 520 full-time employees and structural equation modeling with bootstrapped mediation tests, we examine the structural associations among these constructs. Results indicate that ethical leadership and moral signals are positively associated with emotional energy, which demonstrates the strongest direct association with sustainable wellbeing and significantly mediates the ethical leadership–wellbeing relationship. In contrast, psychological resources exhibit a more conditional and structurally nuanced pattern, suggesting that sustainable wellbeing may depend less on resource accumulation than on the stabilization of emotional expenditure within workplace interactions. By clarifying hierarchical distinctions among affective vitality, adaptive capacity, and contextual moral inputs, this study enhances conceptual precision in ethical leadership research and advances a structured account of sustainable employee wellbeing.

## 1 Introduction

Over the past decade, concerns about employee wellbeing have intensified across both advanced and emerging economies. Even prior to the COVID-19 pandemic, OECD reports documented rising levels of workplace stress and declining work–life balance in several member countries (OECD, 2019). Following the pandemic, global surveys reported heightened emotional exhaustion and disengagement among employees in the United States and Europe (Gallup, 2023). The World Health Organization further highlighted substantial productivity losses linked to workplace anxiety and depression, underscoring the broader economic consequences of sustained psychological strain (World Health Organization, 2022). This pattern aligns with evidence that chronic job stressors, insufficient recovery, and escalating demands may reinforce a “recovery paradox” that undermines long-term wellbeing (Sonnentag, 2018). Taken together, these developments indicate that employee wellbeing represents not a localized organizational concern but a structural challenge confronting contemporary workplaces. Although many organizations have strengthened formal commitments to ethical governance and responsible leadership during this period, persistent emotional instability suggests that ethical commitments alone do not automatically translate into sustainable psychological wellbeing. In response to these challenges, research on ethical leadership has continued to expand, particularly regarding its relevance for employee wellbeing. Meta-analytic evidence indicates that ethical leadership is consistently associated with a broad range of positive employee outcomes, including wellbeing-related indicators (Bedi et al., 2015). Recent scholarship further positions ethical leadership as a cornerstone of organizational integrity, highlighting its relevance in morally demanding environments (Adisa et al., 2025). Empirical findings also suggest that servant leadership relates to employee wellbeing through supportive psychological conditions such as psychological safety (Wang et al., 2022). While these studies underscore the importance of moral leadership, they frequently treat psychological processes as relatively undifferentiated mechanisms, leaving limited clarity regarding how distinct layers of psychological functioning may be structurally organized.

A closer examination of the literature reveals partially fragmented explanatory approaches. One stream foregrounds relational and interactional processes, suggesting that alignment—or misalignment—in leader–member relationships can shape employees’ engagement-related experiences (Matta et al., 2015). Another stream adopts a resource-based perspective, emphasizing that psychological resources can buffer strain and sustain functioning, yet may themselves be shaped by demanding contexts and stress appraisals (Halbesleben et al., 2014). More recent work indicates that authentic leadership may correspond with employee wellbeing through resource-related pathways under job stress, with individual differences such as emotional intelligence influencing these associations (Shih and Yeh, 2024). However, across these perspectives, affective activation and resource capacity are often implicitly treated as interchangeable or sequentially connected, which may obscure potential conceptual and empirical distinctions between short-term emotional vitality and relatively stable adaptive capacity. In parallel, increasing attention has been directed toward the moral context of organizations. Evidence suggests that ethical climate and leadership jointly shape work commitment and broader organizational behavior, underscoring the influence of contextual moral cues on employee responses (Kumar and Ramraj, 2025). Research on perceived corporate social responsibility further indicates that moral self-efficacy and wellbeing-related processes are implicated in employees’ reactions to organizational moral positioning (Song et al., 2024). Studies of exploitative leadership likewise demonstrate that moral cognition and meaning-related processes coexist with emotional experience in shaping employee reactions and ethical silence (Wang et al., 2023). Yet leadership behavior and contextual moral signals are frequently examined as separate explanatory domains rather than as coexisting moral inputs operating within the same analytical structure.

Overall, recent research has generated substantial empirical evidence but has rarely integrated affective vitality, psychological capacity, and contextual moral signals within a unified empirical model. Moreover, reliance on single-mediator explanations may obscure structural differentiation among distinct psychological processes. Without clearly distinguishing between levels of psychological functioning, it remains uncertain whether moral leadership is primarily associated with immediate affective activation, broader adaptive capacity, or contextual moral interpretation. Importantly, given the predominance of cross-sectional designs in this stream of research, caution is warranted in interpreting such associations as causal processes. To address these limitations, the present study advances a differentiated psychological framework. Emotional energy is conceptualized as employees’ perceived affective vitality arising in workplace interactions. Psychological resources are defined as relatively stable adaptive capacities reported by employees. These constructs are treated as analytically distinct components rather than sequential stages of development. In addition, moral signals are incorporated as contextual moral inputs that coexist with ethical leadership within the same empirical model. Given the cross-sectional nature of the data, the study focuses on examining structural associations rather than causal effects. By examining leadership behavior, moral signals, affective vitality, and psychological capacity simultaneously, this study makes three contributions. First, it clarifies the conceptual boundary between short-term affective activation and broader psychological capacity in the ethical leadership literature. Second, it integrates leadership behavior and contextual moral signals within a unified analytical framework. Third, it compares the relative statistical associations of these differentiated mechanisms with sustainable employee wellbeing, offering a more fine-grained understanding of how moral leadership and moral context relate to employee psychological functioning.

## 2 Literature review

### 2.1 Ethical leadership and sustainable employee wellbeing

In organizational contexts, employee wellbeing is expressed through daily affective experiences, yet it reflects broader patterns of psychological functioning shaped by ongoing relational exchanges. Early empirical studies on ethical leadership emphasized its direct association with psychological empowerment and role meaning, suggesting that fairness and responsibility communicated through managerial conduct correspond with positive employee states (Rantika and Yustina, 2017). Subsequent investigations reinforced this view by demonstrating that consistent ethical practices contribute to supportive relational environments linked to reduced psychological strain (Teimouri et al., 2018). More recent evidence further indicates that ethical leadership can foster employees’ psychosocial wellbeing and positive behavioral outcomes by shaping supportive organizational conditions (Islam et al., 2024). As the literature expanded, scholars began to examine employee wellbeing not only as an outcome but also as a relationally embedded process. Research in role-specific and professional contexts suggested that wellbeing functions within broader patterns of engagement and voice behavior under ethical leadership (Ahmad and Al-Shbiel, 2019; Ejaz et al., 2022). More recent studies further highlighted that inclusive or integrity-based leadership contributes to ethical climate and psychological safety, which are statistically associated with lower emotional exhaustion (Li and Peng, 2022). Interaction-focused research also indicates that the strength of the ethical leadership–wellbeing association depends on the quality and consistency of leader–subordinate exchanges. Overall, these developments demonstrate that ethical leadership is consistently associated with employee wellbeing across diverse contexts and relational configurations. However, the psychological architecture underlying this association remains less clearly specified. In particular, prior research often treats affective experiences, empowerment, safety perceptions, and adaptive capacities as closely related indicators of wellbeing without explicitly distinguishing their structural level. This limited differentiation constrains theoretical precision in explaining how ethical leadership corresponds with sustainable employee wellbeing under varying organizational conditions.

### 2.2 Moral signals and the construction of psychological meaning in organizations

Recent organizational research increasingly conceptualizes moral signals as salient contextual cues embedded within ethical climate, justice perceptions, and leadership conduct that shape how employees interpret the legitimacy and moral coherence of their work environment. Empirical evidence suggests that ethical climate is statistically associated with employees’ sense of meaning and wellbeing-related perceptions, positioning moral signals as shared interpretive frameworks rather than merely formal policy expressions (Pandey et al., 2024). Similarly, research demonstrates that ethical climate corresponds with work engagement and job satisfaction, indicating that collective moral norms shape motivational and affective evaluations of work (Ramajoe et al., 2024). Other studies further show that perceptions of ethical climate and organizational justice are linked to employee outcomes through attribution processes, highlighting that moral signals influence how employees cognitively frame leadership effectiveness and organizational intentions (Fein et al., 2023). At the same time, research also documents the destabilizing effects of morally inconsistent or exploitative contexts. Wang et al. (2023) report that exploitative leadership is associated with ethical silence through shifts in work meaningfulness and moral potency, indicating that moral cues can correspond with both cognitive and affective fluctuations. Likewise, Wnuk et al. (2025) demonstrate that ethical climate is statistically associated with organizational cynicism via person–organization match and identification processes, suggesting that moral signals operate through relational and identity-based meaning construction mechanisms.

Across these studies, however, a structural ambiguity becomes apparent. In some frameworks, moral signals are conceptualized as relatively stable contextual background conditions—such as ethical climate or institutional justice—that provide interpretive structure. In others, they are positioned as proximal psychological mechanisms embedded within mediation chains, directly associated with affective states, cognitive appraisals, or wellbeing-related outcomes. This variability in theoretical positioning generates conceptual tension regarding the structural level at which moral signals operate. Moreover, prior research rarely differentiates whether moral signals primarily correspond with immediate emotional activation, identity-based interpretation, or more enduring layers of psychological functioning. Emotional evaluations, attribution processes, relational identification, and wellbeing perceptions are frequently incorporated within the same explanatory sequence without explicit structural differentiation. Such theoretical blending not only obscures the hierarchical positioning of moral signals within ethical leadership frameworks but may also contribute to inconsistent empirical interpretations across studies. Accordingly, the present study conceptualizes moral signals as contextual moral inputs and analytically distinguishes their associations with emotional energy and psychological resources as separate layers of psychological functioning, thereby clarifying their structural role in the architecture of sustainable employee wellbeing.

### 2.3 Emotional energy and psychological resources as distinct psychological layers

Recent organizational research increasingly examines internal psychological mechanisms linking workplace experiences to employee wellbeing; however, these mechanisms are frequently positioned within overlapping explanatory sequences without explicit structural differentiation. A growing stream of scholarship foregrounds affective activation in daily work contexts, emphasizing employees’ momentary vigor, enthusiasm, and emotional activation as salient experiential components of workplace functioning. For example, Zhang et al. (2023) demonstrate that customer mistreatment corresponds with affective rumination and reduced next morning vigor, illustrating how immediate emotional reactions fluctuate in response to interpersonal encounters. Similarly, research on servant leadership indicates that relational safety and wellbeing-related perceptions are embedded within ongoing interactional processes shaping employees’ experiential states (Wang et al., 2022), reinforcing the prominence of short-term affective vitality in contemporary wellbeing research. In parallel, another stream of scholarship emphasizes broader adaptive capacities that structure how employees interpret and navigate demanding work environments. Al Halbusi et al. (2023) report that self-efficacy and individual psychological factors correspond with work engagement, suggesting that relatively stable psychological strengths are embedded within employees’ work-related evaluations. Likewise, Septia Nanda and Nio (2025) show that perceived organizational support is statistically associated with employee wellbeing, positioning support-based perceptions as enduring evaluative capacities rather than transient emotional states. Within this perspective, psychological resources—such as efficacy beliefs, regulatory strength, and perceived support—are conceptualized as relatively stable adaptive structures shaping employees’ interpretations of workplace experiences. Despite these advances, prior studies often incorporate affective vigor, engagement, psychological safety, self-efficacy, and resource-related constructs within the same mediation frameworks without explicitly distinguishing their structural levels. Emotional activation and adaptive capacity are frequently modeled as interchangeable mechanisms, thereby blurring the distinction between immediate experiential states and relatively stable evaluative capacities. Without clarifying this hierarchical differentiation, prior research risks conflating short-term affective vitality with broader resource-based structures, which may obscure the structural specificity of leadership–wellbeing associations and complicate interpretation across empirical findings. This conceptual blending limits theoretical precision in determining whether workplace moral inputs are more closely aligned with momentary emotional energy or with enduring psychological resources. To address this structural ambiguity, the present study analytically differentiates emotional energy defined as employees’ perceived affective vitality arising in workplace interactions from psychological resources, conceptualized as relatively stable adaptive capacities reported by employees, and models these constructs simultaneously yet distinctly in order to clarify their hierarchical positioning within the architecture of sustainable employee wellbeing.

### 2.4 From fragmented mechanisms to a structured process perspective

Recent scholarship consistently demonstrates that morally grounded leadership and ethical organizational environments are statistically associated with employee wellbeing–related outcomes across diverse contexts. Responsible leadership has been framed as a relational orientation that situates moral accountability within leadership processes and employee evaluations (Maak and Pless, 2021), while empirical research on exploitative leadership indicates that moral positioning corresponds with shifts in work meaningfulness and moral potency, underscoring the evaluative sensitivity of employees to leadership-based moral cues (Wang et al., 2023). At the contextual level, organizational ethical climate has been shown to correspond with employees’ sense of meaning and wellbeing-related perceptions, reinforcing the interpretive role of shared moral norms in shaping psychological assessments of work environments (Pandey et al., 2024). Related studies further demonstrate that ethical climate and organizational justice operate through attribution-based cognitive processes influencing employee evaluation patterns (Fein et al., 2023), and that ethical climate is associated with organizational cynicism through relational fit and identification mechanisms, highlighting the identity-based dimension of moral signals (Wnuk et al., 2025). Parallel research emphasizes affective vitality in daily work contexts, showing that interpersonal mistreatment corresponds with fluctuations in next-morning vigor and emotional activation (Zhang et al., 2023), thereby foregrounding short-term experiential states as salient elements of employee functioning. At the same time, scholarship examining individual psychological factors suggests that self-efficacy and related strengths correspond with work engagement, positioning these constructs as relatively stable adaptive capacities embedded in employees’ work-related evaluations (Al Halbusi et al., 2023). Studies of supportive leadership and HR practices similarly indicate that employee wellbeing is associated with contextual support mechanisms operating at multiple organizational levels (Hauff et al., 2020). Taken together, these streams provide substantial empirical insight yet remain structurally heterogeneous, as leadership behavior, moral climate, affective activation, and adaptive capacity are frequently examined within isolated or single-mediator frameworks that do not clarify their hierarchical positioning within the broader psychological architecture of wellbeing. This fragmentation reflects not merely diversity in research design but an unresolved structural ambiguity regarding how different layers of psychological functioning are positioned within ethical leadership research. Without explicitly distinguishing between immediate affective vitality and broader psychological resources, existing models risk conflating experiential states with evaluative capacities, thereby limiting conceptual precision in interpreting how moral leadership and contextual moral signals correspond with sustainable employee wellbeing. A structured process perspective that simultaneously incorporates leadership conduct and moral signals while analytically differentiating emotional energy from psychological resources therefore enhances theoretical coherence and strengthens structural clarity in understanding sustainable employee wellbeing.

### 2.5 Hypotheses development

Building on the literature reviewed above, this study develops five hypotheses linking ethical leadership, moral signals, emotional energy, psychological resources, and sustainable employee wellbeing within a structured conceptual framework. Prior research suggests that ethical leadership shapes employees’ workplace experiences by providing moral guidance, fairness, and behavioral consistency. When leaders demonstrate integrity and ethical role modeling, employees are more likely to perceive their work environment as predictable and normatively coherent. Such relational stability can foster positive emotional activation and engagement in workplace interactions, as employees feel more confident in interpreting organizational expectations and interpersonal dynamics. In morally consistent environments, ethical leadership may therefore enhance employees’ affective vitality and emotional engagement with their work. Accordingly, ethical leadership is expected to be positively associated with employees’ emotional energy (H1).

Beyond leadership behavior, the broader moral environment of organizations may also influence employees’ emotional activation. Moral signals embedded in organizational practices, norms, and expectations provide employees with contextual cues regarding acceptable conduct and organizational values. When these signals are perceived as clear and coherent, employees may experience reduced ambiguity in interpreting workplace interactions and greater alignment with organizational norms. Such contextual clarity may strengthen employees’ psychological comfort and emotional engagement in their work environment. Therefore, moral signals are expected to be positively associated with employees’ emotional energy (H2).

Emotional energy reflects employees’ perceived vitality, enthusiasm, and affective activation in workplace experiences. Research on workplace wellbeing suggests that affective vitality constitutes a key experiential foundation for sustained psychological functioning in organizations. Employees who experience higher levels of emotional energy are more likely to maintain engagement with their work tasks, interact more positively with colleagues, and cope more effectively with workplace demands. These positive emotional dynamics can contribute to a more stable sense of psychological functioning and wellbeing over time. Accordingly, emotional energy is expected to be positively associated with sustainable employee wellbeing (H3).

In addition to momentary emotional activation, employees’ broader adaptive capacities may also contribute to their wellbeing. Psychological resources such as resilience, regulatory capacity, and perceived coping ability enable individuals to manage workplace demands and maintain psychological balance under challenging conditions. Employees who possess stronger psychological resources may therefore experience greater stability in their psychological functioning and report higher levels of sustained wellbeing. Accordingly, psychological resources are expected to be positively associated with sustainable employee wellbeing (H4).

Finally, ethical leadership may also influence employee wellbeing directly beyond the psychological mechanisms discussed above. Ethical leaders foster fairness, trust, and moral clarity in organizational interactions, which may reduce uncertainty and strengthen employees’ perceptions of organizational support. These relational conditions may contribute directly to employees’ psychological stability and positive functioning at work. Therefore, ethical leadership is expected to be positively associated with sustainable employee wellbeing (H5).

## 3 Methods

### 3.1 Research design

This study employed a cross-sectional survey design to examine the relationships among Ethical Leadership, Moral Signals, Emotional Energy, Psychological Resources, and Sustainable Employee wellbeing. Individual employees served as the unit of analysis. The research model investigates how ethical leadership and contextual moral signals relate to employee wellbeing through distinct psychological mechanisms.

### 3.2 Sample and data collection

Data were collected from 500 full-time employees working in various organizations across China. Participation in the survey was voluntary and anonymous, and respondents were informed that their answers would be used solely for academic research purposes. These procedures were implemented to reduce evaluation apprehension and potential social desirability bias. Gender was coded as a binary variable (0 = female, 1 = male) and included as a control variable to examine potential heterogeneity in the relationship between ethical leadership and sustainable well-being.

### 3.3 Measures

All focal constructs were measured using established multi-item self-report scales adapted from prior research. Ethical Leadership was assessed using six items capturing leaders’ fairness, integrity, and ethical role modeling (e.g., “My supervisor sets an example of how to do things the right way in terms of ethics”). Moral Signals were measured with five items reflecting perceived clarity and consistency of organizational moral expectations (e.g., “In my organization, acceptable ethical behavior is clearly communicated”). Emotional Energy was assessed using five items capturing affective vitality and engagement in workplace interactions (e.g., “I feel energized and emotionally engaged when interacting with colleagues at work”). Psychological Resources were measured with four items reflecting perceived regulatory capacity and psychological resilience (e.g., “I am able to effectively manage psychological challenges in my work”). Sustainable wellbeing was assessed using six items capturing relatively stable positive psychological functioning at work rather than temporary affective states (e.g., “I experience a stable sense of psychological wellbeing in my work over time”). All items were rated on a five-point Likert scale ranging from 1 (strongly disagree) to 5 (strongly agree).

### 3.4 Analytical strategy

**Figure 1:**
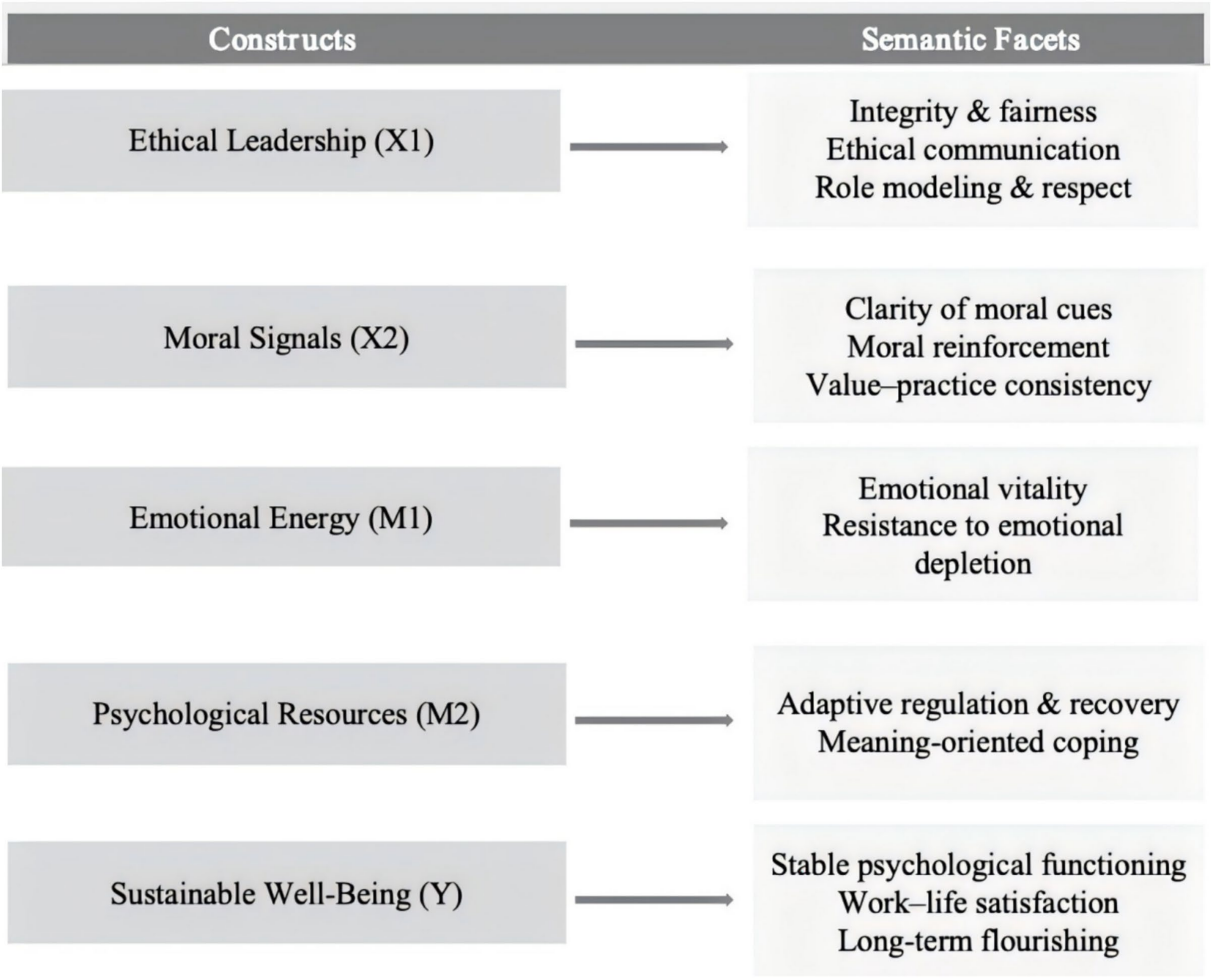
Conceptual measurement framework of the study.

Data analysis proceeded in several stages. First, descriptive statistics and bivariate correlations were calculated to examine preliminary relationships among the study variables. Second, measurement reliability and validity were evaluated using Cronbach’s alpha, composite reliability (CR), average variance extracted (AVE), standardized factor loadings, and discriminant validity criteria based on the Fornell–Larcker approach. Structural relationships were subsequently estimated using structural equation modeling (SEM), which allows the simultaneous estimation of direct and indirect effects within the hypothesized model. Mediation effects were tested using bias-corrected bootstrapping with 2,000 resamples to generate confidence intervals for indirect effects. Variance inflation factors (VIF) were calculated to assess potential multicollinearity. In addition, a group-interaction specification was estimated to examine potential heterogeneity across contextual conditions. All statistical analyses were conducted in Python using pandas for data management, scikit-learn for exploratory procedures, semopy for confirmatory factor analysis and structural equation modeling, statsmodels for regression diagnostics, and matplotlib for visualization. Statistical significance was evaluated at conventional thresholds (*p* < 0.05). Finally, Harman’s single-factor test was performed to assess the potential influence of common method variance (Figure 1).

## 4 Results

### 4.1 Descriptive profile of core constructs

As reported in Table 1, the mean values of all core constructs are slightly below the midpoint of the five-point scale, indicating that employees perceive their organizational ethical environment and psychological functioning as moderate rather than strongly reinforced. Ethical Leadership (*M* = 2.872, SD = 0.923) and Moral Signals (*M* = 2.874, SD = 0.934) suggest that ethical guidance and moral cues are present but not consistently salient across daily work interactions. Similarly, Emotional Energy (*M* = 2.877, SD = 0.955), Psychological Resources (*M* = 2.889, SD = 0.961), and Sustainable wellbeing (*M* = 2.861, SD = 0.916) cluster at comparable levels, reflecting that affective vitality, accumulated psychological capacity, and enduring wellbeing are observable but not deeply consolidated within the sample. The relatively large standard deviations (ranging from 0.916 to 0.961) indicate substantial dispersion in employees’ experiences, with minimum values reaching the lower boundary of the scale and maximum values reaching the upper boundary. This pattern demonstrates that organizational ethical practices and psychological states are not uniformly distributed but vary considerably across individuals and contexts. The coexistence of moderate averages and wide dispersion suggests an environment characterized by fluctuating moral clarity and differentiated emotional engagement rather than uniformly strong or uniformly weak ethical conditions. Psychological Resources, in particular, do not appear to exhibit a pronounced accumulation advantage, remaining sensitive to situational dynamics and potentially contingent on sustained organizational support. Sustainable wellbeing mirrors this moderate profile, implying that positive psychological functioning continues to depend on ongoing contextual inputs rather than having stabilized into a resilient personal baseline. Regarding control variables, Control 1 (*M* = 35.10, SD = 8.593) spans from 20 to 49, covering early to mid-career stages and thereby capturing meaningful variation in professional experience, while the binary controls (Control 2 and Group) are approximately evenly distributed, reducing concerns of structural imbalance. Taken together, the descriptive statistics indicate adequate variability, absence of ceiling or floor effects, and balanced sample composition, thereby providing a sound empirical foundation for subsequent reliability, validity, and structural model analyses.

**Table 1:** Descriptive statistics of study variables.

Variable	Mean	SD	Min	Max
X1_Ethical leadership	2.872	0.923	1	5
X2_Moral signals	2.874	0.934	1	5
M1_Emotional energy	2.877	0.955	1	5
M2_Psychological resources	2.889	0.961	1	5
Y_Sustainable wellbeing	2.861	0.916	1	5
Control 1	35.1	8.593	20	49
Control 2	0.516	0.5	0	1
Group	0.49	0.5	0	1

### 4.2 Measurement quality: internal consistency of multi-item scales

Table 2 provides comprehensive evidence supporting the reliability, convergent validity, discriminant validity, and methodological robustness of the measurement model. Cronbach’s *α* coefficients range from 0.822 to 0.871, exceeding the conventional threshold of 0.70 and indicating satisfactory internal consistency without suggesting problematic item redundancy. Composite reliability (CR) values range from 0.839 to 0.896, further confirming scale stability. All average variance extracted (AVE) values exceed the recommended cutoff of 0.50, demonstrating adequate convergent validity, while standardized factor loadings range from 0.68 to 0.83 and remain well above the minimum acceptable level of 0.60. Together, these results indicate that individual indicators contribute substantively to their respective latent constructs and that the measurement structure is psychometrically sound. Importantly, none of the constructs exhibit excessively high reliability coefficients (e.g., α > 0.95), which could otherwise indicate inflated covariance attributable to method effects rather than substantive content. Discriminant validity analysis further demonstrates that the square roots of AVE for all constructs exceed the corresponding inter-construct correlations, confirming empirical distinctiveness among Ethical Leadership, Moral Signals, Emotional Energy, Psychological Resources, and Sustainable wellbeing. This pattern of results indicates that the constructs are not manifestations of a single overarching response tendency but represent theoretically differentiated dimensions. To directly evaluate the potential influence of common method variance, Harman’s single-factor test was conducted using all 26 measurement items. The first unrotated factor accounted for 35.36% of the total variance, substantially below the 50% threshold typically used to indicate serious common method bias. This finding suggests that no single factor dominates the covariance structure. Moreover, the subsequent structural equation modeling results reveal both positive and negative significant paths of varying magnitudes, further reducing the likelihood that the observed associations are artifacts of a uniform response style or common method inflation, which would typically produce uniformly positive and systematically elevated relationships. The combination of acceptable reliability coefficients, adequate convergent validity, clear discriminant validity, moderate inter-construct correlations, a sub-threshold single-factor variance, and differentiated structural path directions provides converging evidence that common method variance does not materially threaten the validity of the findings. Collectively, these results establish a rigorous psychometric foundation for the structural analyses and support the interpretability of the hypothesized mechanism pathways.

**Table 2:** Measurement reliability and convergent validity.

Construct	Items	Cronbach’s α	CR	AVE	Std. Loadings
Ethical leadership (EL)	6	0.871	0.896	0.591	0.69–0.83
Moral signals (MS)	5	0.822	0.839	0.511	0.68–0.78
Emotional energy (EE)	5	0.853	0.872	0.577	0.72–0.81
Psychological resources (PR)	4	0.829	0.858	0.602	0.73–0.81
Sustainable wellbeing (WB)	6	0.860	0.886	0.565	0.71–0.82

### 4.3 Preliminary structural associations among core constructs

Table 3 reports the bivariate correlations among the core constructs. Ethical Leadership is positively associated with Moral Signals (*r* = 0.524, *p* < 0.001), indicating related yet empirically distinguishable moral inputs. Both Ethical Leadership (*r* = 0.603, *p* < 0.001) and Moral Signals (*r* = 0.526, *p* < 0.001) show significant positive correlations with Emotional Energy, suggesting that leadership conduct and contextual moral cues are closely aligned with employees’ affective vitality. Emotional Energy demonstrates the strongest association with Sustainable wellbeing (*r* = 0.556, *p* < 0.001), followed by Moral Signals (*r* = 0.505, *p* < 0.001) and Ethical Leadership (*r* = 0.486, *p* < 0.001). All positive correlations remain moderate in magnitude, supporting construct separability. In contrast, Psychological Resources exhibit a differentiated pattern, showing negative correlations with Ethical Leadership (*r* = −0.250, *p* < 0.001), Moral Signals (*r* = −0.250, *p* < 0.001), and Emotional Energy (*r* = −0.150, *p* < 0.001), while remaining unrelated to Sustainable wellbeing at the bivariate level (*r* = 0.010, ns). The coexistence of positive and negative associations indicates structural differentiation rather than uniform covariance among variables. Overall, the correlation matrix provides preliminary support for the hypothesized relationships and informs the subsequent structural equation modeling analyses.

**Table 3:** Means, standard deviations, and correlations among core constructs.

Variable	1	2	3	4	5
1. X1_Ethical leadership	1.0				
2. X2_Moral signals	0.524***	1.0			
3. M1_Emotional energy	0.603***	0.526***	1.0		
4. M2_Psychological resources	−0.250***	−0.250***	−0.15***	1.0	
5. Y_Sustainable wellbeing	0.486***	0.505***	0.556***	0.01	1.0

***p < 0.001.

### 4.4 Structural model estimates

**Figure 2:**
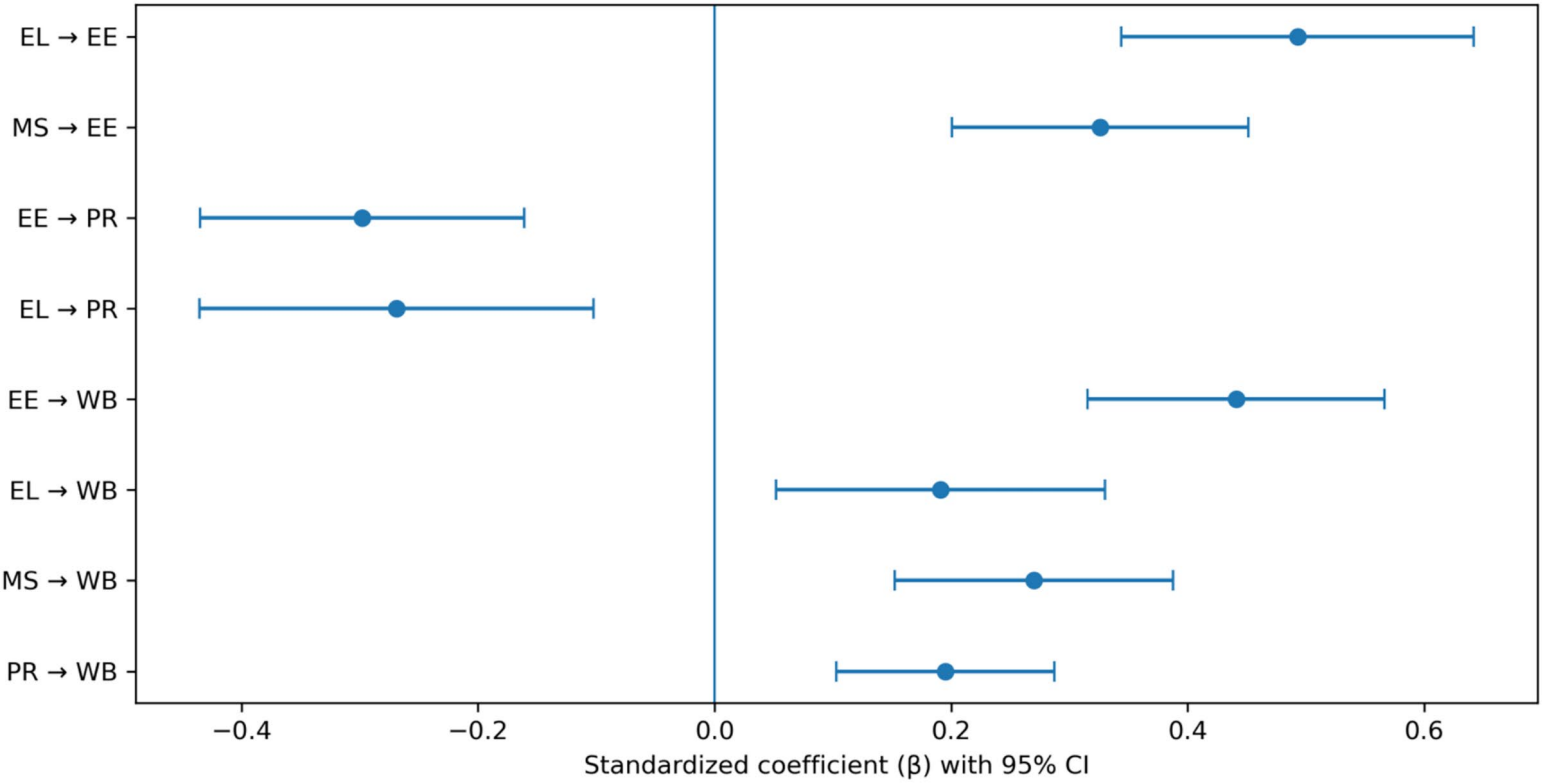
SEM path coefficients predicting sustainable wellbeing.

Table 4 reports the standardized structural path estimates for the hypothesized model, and Figure 2 visually presents the corresponding coefficients with 95% confidence intervals. Prior to estimating the structural relationships, confirmatory factor analysis indicated that the measurement model demonstrated an acceptable fit to the data (CFI = 0.94, TLI = 0.93, RMSEA = 0.052, SRMR = 0.047), supporting the adequacy of the latent measurement structure. Consistent with the correlational pattern observed in Table 3, Ethical Leadership (*β* = 0.493, *p* < 0.001) and Moral Signals (*β* = 0.326, *p* < 0.001) are positively associated with Emotional Energy when estimated simultaneously within the structural model, indicating that organizational moral inputs remain robust predictors of affective activation after accounting for shared variance. In contrast, both Emotional Energy (*β* = −0.298, *p* < 0.001) and Ethical Leadership (*β* = −0.269, *p* < 0.001) display negative associations with Psychological Resources, replicating the differentiated relational profile observed at the bivariate level and suggesting that heightened emotional engagement and stronger ethical expectations may correspond to increased regulatory demands. This pattern also reflects a suppression configuration in which the statistical control of shared variance among predictors reveals the conditional contribution of psychological resources within the broader model structure. With respect to Sustainable wellbeing, Emotional Energy demonstrates the strongest positive structural effect (*β* = 0.441, *p* < 0.001), followed by Moral Signals (*β* = 0.270, *p* < 0.001), Psychological Resources (*β* = 0.195, *p* < 0.001), and Ethical Leadership (*β* = 0.191, *p* = 0.005). As illustrated in Figure 2, none of the confidence intervals cross zero, indicating that all estimated paths are statistically reliable. The magnitudes of the standardized coefficients remain moderate rather than excessive, and the coexistence of positive and negative significant paths further suggests that the structural relationships are differentiated rather than uniformly inflated. This pattern also reduces concerns that the observed associations are driven by a generalized response tendency or common method bias. Overall, the structural estimates extend the preliminary correlational findings and provide empirical support for the proposed mechanism linking organizational moral inputs, emotional activation, psychological resources, and sustainable wellbeing.

**Table 4:** Structural path estimates for the hypothesized model.

Path	*β* (Std.)	SE	*z*	*p*
EL → EE	0.493	0.076	7.962	<0.001
MS → EE	0.326	0.064	5.586	<0.001
EE → PR	−0.298	0.07	−4.011	<0.001
EL → PR	−0.269	0.085	−3.62	<0.001
EE → WB	0.441	0.064	5.91	<0.001
EL → WB	0.191	0.071	2.791	0.005
MS → WB	0.27	0.06	4.243	<0.001
PR → WB	0.195	0.047	3.758	<0.001

Standardized coefficients (β) are reported.

### 4.5 Mediation effect of emotional energy

**Figure 3:**
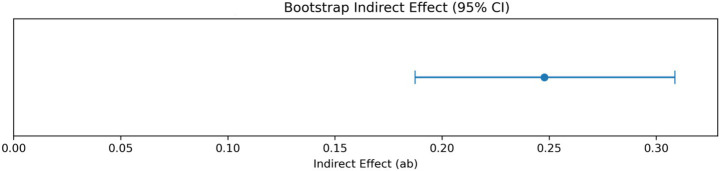
Bootstrap indirect effect of emotional energy.

Results reported in Table 5, and visually illustrated in Figure 3, indicate a statistically significant indirect effect of Ethical Leadership on Sustainable wellbeing through Emotional Energy. The bootstrapped indirect effect based on 2,000 resamples is 0.2478, with a 95% confidence interval ranging from 0.1875 to 0.3087. Because the confidence interval does not include zero, the mediating effect is statistically supported. As shown in Figure 3, the point estimate lies clearly above the zero reference line, and the entire confidence interval remains within the positive range, reinforcing the robustness of the mediation result. The magnitude of the indirect effect is moderate and consistent with the structural coefficients reported in Table 4, suggesting that Emotional Energy functions as a meaningful transmission mechanism linking organizational moral inputs to wellbeing outcomes. Specifically, Ethical Leadership appears to enhance employees’ affective vitality, which in turn contributes to higher levels of Sustainable wellbeing. The mediation result extends the preliminary correlational findings and structural path estimates by demonstrating that part of the influence of Ethical Leadership operates indirectly through Emotional Energy. The statistically differentiated pattern of direct and indirect effects further supports the interpretability of the hypothesized mechanism and reduces the likelihood that the observed mediation is attributable to common method variance or uniform response tendencies. Overall, the bootstrap analysis provides converging evidence for the proposed affective pathway from ethical leadership to sustainable employee wellbeing.

**Table 5:** Bootstrap mediation analysis.

Path (IV → Mediator → DV)	Indirect effect	95% CI lower	95% CI upper	Bootstraps	Sig.
X1_Ethical leadership → M1_Emotional energy → Y_Sustainable wellbeing	0.2478	0.1875	0.3087	2000	***

***p < 0.001.

### 4.6 Multicollinearity diagnostics

Table 6 reports the variance inflation factors (VIF) for the four structural predictors. The observed values range from 1.272 to 1.837, with Ethical Leadership (VIF = 1.819) and Emotional Energy (VIF = 1.837) showing the highest values, followed by Moral Signals (VIF = 1.526) and Psychological Resources (VIF = 1.272). All VIF values are substantially below commonly cited multicollinearity thresholds (e.g., 5.0 or the more conservative 3.0 criterion), indicating that multicollinearity does not pose a threat to parameter stability. Although Ethical Leadership and Emotional Energy exhibit comparatively higher VIF values, their levels remain well within acceptable limits, suggesting moderate shared variance without redundancy. The differentiated pattern of VIF estimates further supports construct separability, as no predictor demonstrates excessive overlap with the others. Taken together, the VIF results indicate that the structural coefficients reported in Table 4 are unlikely to be distorted by multicollinearity and can be interpreted as substantively meaningful associations rather than artifacts of statistical redundancy.

**Table 6:** Variance inflation factors.

Predictor	VIF
X1_Ethical leadership	1.819
M1_Emotional energy	1.837
X2_Moral signals	1.526
M2_Psychological resources	1.272

### 4.7 Moderating role of gender in the ethical leadership–wellbeing relationship

Table 7 reports the estimates from the group-interaction specification. Ethical Leadership remains positively associated with Sustainable wellbeing (*B* = 0.2872, SE = 0.0559, *p* < 0.001), and Moral Signals also exhibit a significant positive effect (*B* = 0.3410, SE = 0.0425, *p* < 0.001), indicating that the primary organizational moral inputs retain their explanatory power in the interaction model. In contrast, Psychological Resources do not demonstrate a statistically significant direct effect in this specification (*B* = 0.0604, SE = 0.0387, *p* = 0.1193). The main effect of Group is negative but not statistically significant (*B* = −0.3139, SE = 0.2233, *p* = 0.1606), suggesting that baseline levels of Sustainable wellbeing do not differ systematically across groups. Importantly, the interaction term between Ethical Leadership and Group is also non-significant (*B* = 0.0914, SE = 0.0739, *p* = 0.2168), indicating that the strength of the relationship between Ethical Leadership and Sustainable wellbeing does not vary meaningfully across group membership. The absence of significant interaction effects suggests that the structural associations identified in the main model are relatively stable across groups and that the positive contribution of ethical leadership to wellbeing operates in a comparable manner across contextual conditions. Overall, the group-based specification does not provide evidence of substantial heterogeneity in the focal structural relationships, reinforcing the robustness of the primary model estimates.

**Table 7:** Group interaction model.

Predictor	*B*	SE	*t*	*P*
EL	0.2872	0.0559	5.1403	0
Group	−0.3139	0.2233	−1.4053	0.1606
MS	0.341	0.0425	8.0259	0
PR	0.0604	0.0387	1.5603	0.1193
EL × Group	0.0914	0.0739	1.2367	0.2168

## 5 Discussion

### 5.1 Ethical leadership and sustainable wellbeing: convergence with prior evidence

The present findings indicate that ethical leadership is positively associated with sustainable employee wellbeing, reinforcing recent evidence that ethical conduct in leadership roles supports psychological functioning and workplace sustainability (Pandey et al., 2024). While prior studies have frequently linked ethical leadership to engagement, meaning, and psychological safety, the current results suggest that its relevance extends beyond attitudinal endorsement toward a more stable form of psychological continuity (Wang et al., 2023). This interpretation is consistent with research demonstrating that leadership behaviors structure employees’ daily emotional experiences rather than merely shaping retrospective evaluations (Zhang et al., 2023). In line with emerging discussions on ethical climate and relational stability, ethical leadership may reduce uncertainty and perceived arbitrariness in organizational interactions, thereby contributing to a more predictable work environment (Fein et al., 2023). From a conservation of resources perspective, contextual stability and reduced ambiguity are central conditions for maintaining psychological resources over time (Hobfoll et al., 2018). Ethical leadership, originally conceptualized as normatively appropriate conduct enacted through interpersonal relationships (Brown and Treviño, 2006), may therefore relate to sustainable wellbeing by stabilizing interaction expectations rather than by solely activating moral identity processes. Recent work further suggests that leadership-driven ethical climates influence employee psychological functioning through ongoing relational regulation rather than isolated value signaling (Wnuk et al., 2025). Taken together, our findings converge with contemporary literature while extending it by positioning ethical leadership as a structural regulator of emotional demand patterns associated with enduring wellbeing in organizational settings (Ramajoe et al., 2024).

### 5.2 Emotional energy as a proximal mechanism: extending resource-based explanations

A dominant assumption in moral leadership research is that wellbeing improvements primarily emerge through the accumulation of psychological resources such as empowerment, engagement, or self-efficacy (Lemoine et al., 2019). However, the present findings complicate this assumption by indicating that emotional energy occupies a more immediate explanatory position than accumulated psychological capital. Recent work distinguishing between stable between-person effects and dynamic within-person processes suggests that leadership influences on wellbeing unfold through rapidly fluctuating emotional mechanisms rather than solely through gradually built cognitive resources (Rudolph et al., 2022). This perspective aligns with scholarship emphasizing that employees actively regulate their wellbeing through momentary emotional processing and reflection, implying that affective dynamics may be more proximal to sustained functioning than structural resource stocks (Ilies et al., 2024). From a conservation of resources framework, resource preservation depends not only on gain cycles but also on preventing ongoing resource loss episodes that silently erode functioning over time (Hobfoll et al., 2018). The present results are consistent with a loss-prevention interpretation in which ethical leadership may relate to sustainable wellbeing by stabilizing emotional expenditure rather than by directly increasing resource accumulation. Furthermore, recent integrative reviews caution against construct redundancy in leadership research, arguing that many leadership constructs overlap conceptually and may obscure distinct psychological mechanisms (Banks et al., 2018). In this light, positioning emotional energy as a proximal mechanism introduces needed differentiation within the moral leadership domain, highlighting that affective regulation processes may operate independently from broader moral framing narratives. Finally, organizational process research suggests that system stability often depends on regulating ongoing micro-level disturbances rather than on building additional structural capacity (Lei and Naveh, 2023). Taken together, the current findings introduce theoretical tension into prevailing accumulation-based explanations by suggesting that sustainable wellbeing may hinge more on managing emotional fluctuations than on expanding psychological resource reserves.

### 5.3 Resource mobilization and recovery imbalance: a conditional perspective

These findings carry important practical implications for organizational leaders who often equate wellbeing enhancement with expanding formal support programs or increasing developmental resources. Contemporary evidence suggests that interventions focused solely on resource provision may overlook the emotional expenditure embedded in everyday interaction processes (Ilies et al., 2024). The present interpretation implies that managerial efforts to improve sustainable wellbeing should prioritize stabilizing decision routines, clarifying role expectations, and reducing unpredictability in interpersonal exchanges, as micro-level disturbances can accumulate into larger psychological strain over time (Lei and Naveh, 2023). Rather than emphasizing resource expansion alone, leaders may need to monitor the rhythm of resource mobilization and recovery cycles within their teams. Conservation of resources theory highlights that preventing resource loss is often more critical than promoting resource gain (Hobfoll et al., 2018), suggesting that minimizing avoidable emotional depletion may produce more durable wellbeing outcomes than introducing additional motivational initiatives. Furthermore, recognizing emotional energy as a frontline regulatory mechanism invites organizations to reconsider performance management systems that intensify accountability without parallel recovery structures. In this sense, sustainable wellbeing may depend less on increasing employees’ psychological capital and more on redesigning organizational processes to reduce chronic emotional overactivation. These insights extend beyond ethical leadership research by encouraging a shift from accumulation-based intervention logic toward stabilization-based organizational design.

## 6 Conclusion, limitations, and future directions

This study set out to clarify how ethical leadership and contextual moral signals are associated with sustainable employee wellbeing by differentiating between distinct layers of psychological functioning. Rather than treating affective vitality and psychological resources as interchangeable mechanisms, we analytically separated emotional energy from adaptive resource capacity and examined them simultaneously within a structured empirical model. The empirical findings provide support for the hypothesized relationships among the focal constructs. Specifically, ethical leadership is positively associated with employees’ emotional energy (supporting H1), and moral signals likewise demonstrate a positive association with emotional energy (supporting H2). Emotional energy, in turn, shows the strongest positive association with sustainable employee wellbeing (supporting H3), while psychological resources also contribute positively to wellbeing outcomes (supporting H4). In addition, ethical leadership maintains a direct positive association with sustainable employee wellbeing beyond these mechanisms (supporting H5). Taken together, these results indicate that emotional energy operates as a more proximal experiential interface through which moral inputs are translated into wellbeing outcomes, whereas psychological resources exhibit a more conditional and structurally nuanced association with sustainable employee wellbeing. By integrating leadership behavior and contextual moral signals within the same analytical structure, this study addresses fragmentation in prior research that frequently examined these inputs in isolation. Modeling both domains concurrently suggests that moral leadership and moral context coexist within a shared psychological architecture rather than forming sequential or redundant explanatory chains. In doing so, the study advances greater conceptual precision within ethical leadership research, responds to calls for stronger construct differentiation, and contributes to a more nuanced understanding of how moral inputs shape enduring psychological functioning in contemporary workplaces.

Several limitations warrant careful consideration. First, the cross-sectional design precludes temporal sequencing and causal inference. Although structural equation modeling and bootstrapped indirect effect estimates provide statistical support for differentiated associations, longitudinal, experience-sampling, and multi-wave designs are necessary to examine dynamic activation and recovery cycles more directly. Second, while reliability diagnostics, discriminant validity tests, and Harman’s single-factor results suggest that common method variance is unlikely to fully account for the observed patterns, the use of self-reported measures invites future replication using multi-source or time-lagged data. Third, the complex statistical profile of psychological resources underscores the need for additional research disentangling between-person stability from within-person mobilization processes. Future research may therefore benefit from adopting temporal and multi-level designs that capture fluctuations in emotional energy alongside longer-term shifts in resource capacity. Experimental or quasi-experimental studies could further clarify boundary conditions under which moral leadership is most strongly associated with emotional stabilization rather than resource accumulation. Cross-cultural comparisons may also illuminate whether the structural differentiation observed here generalizes across institutional contexts with varying moral expectations and organizational norms.

In increasingly demanding and morally complex work environments, ethical leadership appears to relate to employee wellbeing not solely through symbolic moral framing, but through structuring interactional predictability and regulating emotional expenditure. By repositioning emotional energy as a frontline regulatory mechanism and reframing psychological resources as conditionally mobilized capacities, this study offers a more structured and theoretically coherent account of sustainable employee wellbeing. Such differentiation enhances conceptual clarity, strengthens methodological rigor, and provides a foundation for future research aimed at understanding how moral leadership and moral context shape enduring psychological functioning in organizations.

## Data Availability

The raw data supporting the conclusions of this article will be made available by the authors, without undue reservation.
